# A Versatile Microfluidic Device System that Lacks a Synthetic Extracellular Matrix Recapitulates the Blood–Brain Barrier and Dynamic Tumor Cell Interaction

**DOI:** 10.3390/bioengineering11101008

**Published:** 2024-10-10

**Authors:** Daniel Santillán-Cortez, Andrés Eliú Castell-Rodríguez, Aliesha González-Arenas, Juan Antonio Suárez-Cuenca, Vadim Pérez-Koldenkova, Denisse Añorve-Bailón, Christian Gabriel Toledo-Lozano, Silvia García, Mónica Escamilla-Tilch, Paul Mondragón-Terán

**Affiliations:** 1 Laboratorio de Medicina Regenerativa e Ingeniería de Tejidos, Centro Médico Nacional ‘20 de Noviembre’, Instituto de Seguridad y Servicios So Ciales para los Trabajadores del Estado, San Lorenzo 502, 3er Piso. Col. Del Valle, Del. Benito Juárez, Mexico City 03100, Mexico; 2 Laboratorio de Medicina Regenerativa e Inmunoterapia Experimental, Departamento de Biología Celular y Tisular, Facultad de Medicina-Universidad Nacional Autónoma de México, Ciudad Universitaria, Mexico City 04510, Mexico; castell@unam.mx; 3 Departamento Medicina Genómica y Toxicología Ambiental, Instituto de Investigaciónes Biomédicas-Universidad Nacional Autónoma de México, Circuito de la Investigación Científica, Ciudad Universitaria, Coyoacán, Mexico City 04510, Mexico; alieshag@iibiomedicas.unam.mx; 4 Experimental Metabolism and Clinical Research Laboratory, Clinical Research Department, Division of Biomedical Research, Centro Médico Nacional ‘20 de Noviembre’, Instituto de Seguridad y Servicios Sociales para los Trabajadores del Estado, Mexico City 03229, Mexico; suarej05@gmail.com; 5 Laboratorio Nacional de Microscopía Avanzada, Centro Médico Nacional Siglo XXI, Instituto Mexicano del Seguro Social, Mexico City 06720, Mexico; 6 Subdireccion de Investigacion, Centro Médico Nacional ‘20 de Noviembre’—ISSSTE, San Lorenzo 502, 2do Piso. Col. Del Valle, Del. Benito Juárez, Mexico City 03100, Mexico; 7 Coordinación de Investigación, Centro Médico Nacional ‘20 de Noviembre’, Instituto de Seguridad y Servicios Sociales para los Trabajadores del Estado, Mexico City 03229, Mexico; 8 Laboratorio de Inmunogenética, Centro Médico Nacional ‘20 de Noviembre’, Instituto de Seguridad y Servicios Sociales para los Trabajadores del Estado, Mexico City 03229, Mexico; met171179@hotmail.com; 9 Centro de Investigación en Ciencia Aplicada y Tecnología Avanzada Unidad Morelos, Instituto Polítecnico Nacional, Boulevard de la Tecnología, 1036 Z-1, P 2/2, Atlacholoaya 62790, Mexico

**Keywords:** microfluidic system, glioblastoma, tumoral biology, organoid culture

## Abstract

Microfluidic systems offer controlled microenvironments for cell-to-cell and cell-to-stroma interactions, which have precise physiological, biochemical, and mechanical features. The optimization of their conditions to best resemble tumor microenvironments constitutes an experimental modeling challenge, particularly regarding carcinogenesis in the central nervous system (CNS), given the specific features of the blood–brain barrier (BBB). Gel-free 3D microfluidic cell culture systems (gel-free 3D-mFCCSs), including features such as self-production of extracellular matrices, provide significant benefits, including promoting cell–cell communication, interaction, and cell polarity. The proposed microfluidic system consisted of a gel-free culture device inoculated with human brain microvascular endothelial cells (HBEC5i), glioblastoma multiforme cells (U87MG), and astrocytes (ScienCell 1800). The gel-free 3D-mFCCS showed a diffusion coefficient of 4.06 × 10^−9^ m^2^·s^−1^, and it reconstructed several features and functional properties that occur at the BBB, such as the vasculogenic ability of HBEC5i and the high duplication rate of U87MG. The optimized conditions of the gel-free 3D-mFCCS allowed for the determination of cellular proliferation, invasion, and migration, with evidence of both physical and biochemical cellular interactions, as well as the production of pro-inflammatory cytokines. In conclusion, the proposed gel-free 3D-mFCCSs represent a versatile and suitable alternative to microfluidic systems, replicating several features that occur within tumor microenvironments in the CNS. This research contributes to the characterization of microfluidic approaches and could lead to a better understanding of tumor biology and the eventual development of personalized therapies.

## 1. Introduction

The blood–brain barrier (BBB) is a selective barrier that controls the movement of substances between the bloodstream and the brain. Its dysfunction can affect flow dynamics, immune cell entry into the central nervous system (CNS), and interstitial clearance, leading to several neurological diseases, such as Alzheimer’s disease, depression, cognitive impairment, CNS infection, epilepsy, and brain tumors [1,2,3,4,5,6]. Additionally, BBB alterations complicate drug delivery to the CNS, limiting drugs’ effectiveness [6].

The function of the BBB is particularly important in the context of brain tumors, which account for 2–4% of all malignant neoplasms. These tumors are highly heterogeneous and are shaped by genomic alterations and their biological environments. Tumor cells interact dynamically with glial, immune, and endothelial cells and extracellular matrices within complex 3D structures [7,8,9,10,11]. Cancer stem cells (CSCs) in these tumors exhibit characteristics that are similar to those of normal stem cells and express several markers, including CD133+, CD15, CD49f+, CD36+, A2B5+, CD44+, L1CAM+, and the epidermal growth factor receptor (EGFR+). In addition, several transcription factors also play an important role in the identification of CSC subpopulations, including BMI1+, Olig2+, Oct3/4, SOX2+, NESTIN, MYC, and IDH1 [12,13,14,15,16].

**Figure 1:**
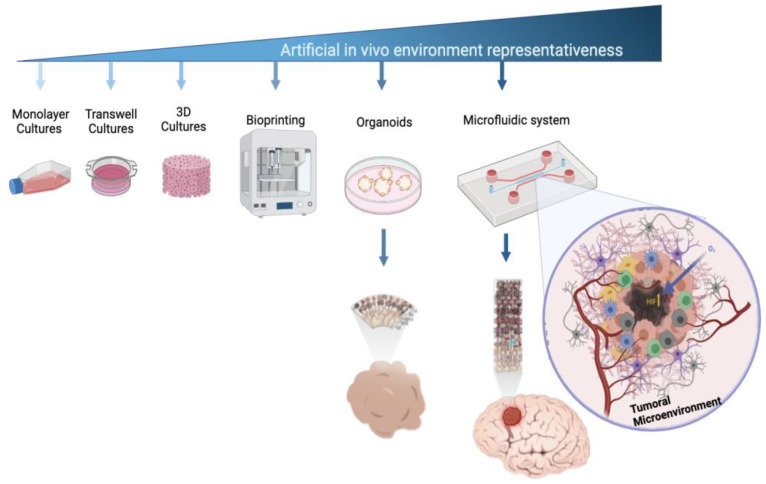
Cell culture models used to mimic the microenvironmental complexity of brain tumors. Cell culture platforms represent the increasing complexity of cellular models that are used to study brain tumors. This complexity is related to their ability to mimic real biological microenvironments, 3D architectures, and cell-to-cell and micro-vascularity interactions.

Developing effective therapies requires an in-depth understanding of cellular and molecular mechanisms, especially those that affect tumor microenvironments [17,18]. While 3D cell culture techniques mimic tumor physiology better than 2D models, they still fall short of replicating the complexity of the multicellularity and vascularization necessary for tumor growth and migration [19,20]. Advances in microfluidic cell culture systems have offered more accurate models of human tissue complexity (Figure 1), enabling the study of tumor formation, progression, and therapeutic responses [20,21,22,23].

The blood–brain barrier (BBB) is a highly regulated structure in which fluid–cell permeability and interactions are tightly controlled. Microfluidic devices are valuable models that can replicate the complexity of BBB tumor microenvironments and offer several advantages, such as mimicking pathophysiological conditions with low sample volumes and high-throughput screening [24]. These devices are particularly useful in glioblastoma research that studies tumor environments, cell migration, drug screening, and therapeutic responses [25,26].

Various microfluidic models have been developed to study BBB characteristics, such as using immortalized rat brain endothelial cells to explore the BBB microvasculature. These models allow for long-term culturing and real-time monitoring, inducing tight junction formation and transporter functionality [26]. Humanized versions, which incorporate human brain endothelial cells and astrocytes, have been used to further explore the effects of shear stress, inflammation, and drug transport mechanisms across the BBB [27,28].

Likewise, humanized microfluidic devices, constituting primary brain-derived microvascular endothelial cells that are in contact with cultured pericytes and astrocytes, have been useful for studying neuroinflammatory processes at the BBB, the secretion of pro-inflammatory cytokines like granulocyte colony-stimulating factor (G-CSF) and interleukin-6 (IL-6) [29]), the consequent local metabolic changes [30], and the pharmacokinetics and pharmacodynamics of drugs within the central nervous system [31], including the mechanisms of opioid transport across the BBB [32]. Other humanized microfluidic systems have further evaluated other effects such as hypoxia. Using cell complexes formed of pluripotent stem cell-derived human brain microvascular endothelium, and primary brain astrocytes and pericytes, it has been possible to explore the effects of hypoxia on brain cellular differentiation [33]. Furthermore, CNS metastasis, transcytosis, and the permeability of molecules and extracellular vesicles across the BBB have been studied by adding breast cancer cells to the previously described devices [34]. Microfluidic systems enable the precise control of cell–cell interactions, while embedded ECM supports cell–microenvironment interactions, survival, differentiation, migration, and drug permeability. However, challenges with these systems include the use of varying ECMs, which may limit natural in vivo processes such as cell polarity development and vasculogenesis, as well as the specialized structures and responses that are triggered by self-produced ECM [35]. Other limitations include potential contamination during cell loading, variability in hydrogel composition, and uneven ECM distribution, which can affect oxygen and nutrient transport and alter flow patterns [36]. Gel-free 3D microfluidic systems (gel-free 3D-mFCCSs) offer a solution by promoting cell–cell communication, gap junction formation, and cell polarity restoration without synthetic ECM. These systems are particularly well suited for studying solid tumor models via spheroid formation [34]. This study aimed to develop a gel-free 3D-mFCCS that supports multiple cell types, such as endothelial cells and glioblastoma cells, to mimic the blood–brain barrier in CNS tumor development. 

## 2. Materials and Methods

**Figure 2:**
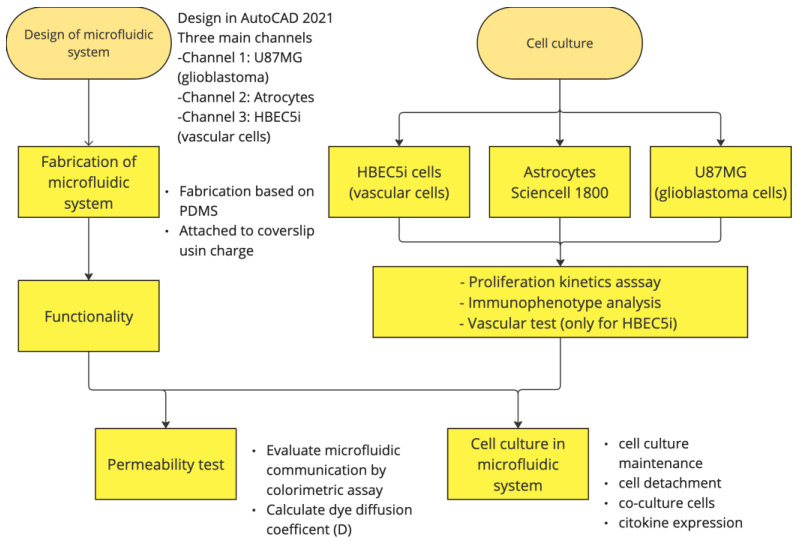
Design and fabrication of the microfluidic system for cellular interaction research.

The project was approved by the ethics, research, and biosafety committees of the Centro Médico Nacional ‘20 de Noviembre’, Institute of Social Security and Services for State Workers (ISSSTE; approval no. 468.2020). Human U87MG cells; glioblastoma multiforme from the American Type Culture Collection (ATCC, Manassas, VA, USA), with EMEM +10% fetal bovine serum [2], Merck/Millipore Sigma Burlington, MA, USA), HBEC5i (from the ATCC, with DMEM/F12 +10% fetal bovine serum, made in Manassas, Virginia USA), and astrocytes from ScienCell, Carlsbad, CA, USA (catalog number 1800, with supplement culture medium 1801) were utilized, following the specifications described in the technical datasheet, All cell lines were maintained at 80% confluence for cell subculturing, and all experiments were conducted with subcultures of fewer than 8 subcultures. Figure 2 shows the general process carried out in this study.

### 2.1. Design, Fabrication, and Permeability Testing of the Microfluidic System

**Figure 3:**
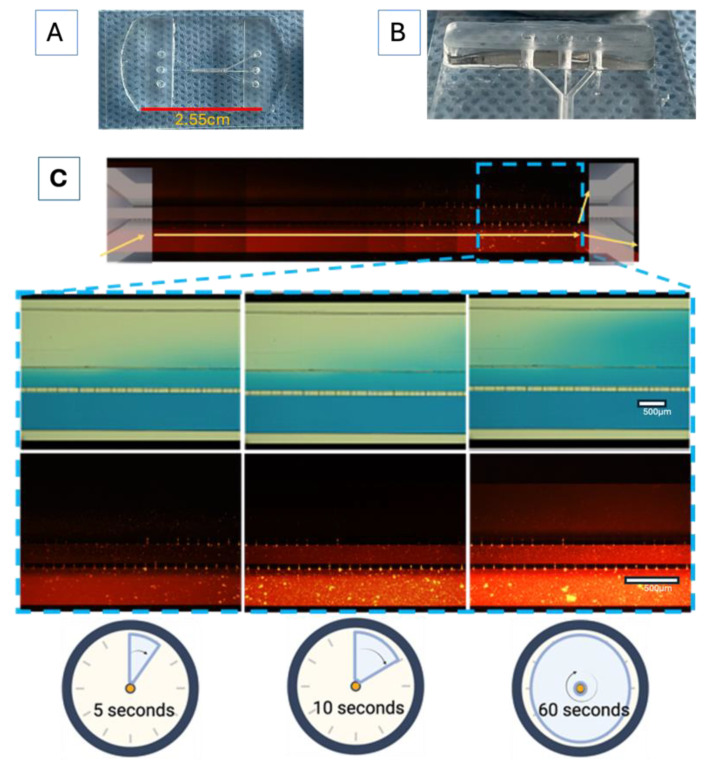
The device design and permeability of the proposed microfluidic system: (**A**) comparative size of device; (**B**) lateral view of device; (**C**) progressive permeability in the device, demonstrated by the permeation of blue food stain (upper panel) and Texas red dextran (10WD; lower panel). Time is also indicated for comparison. The yellow arrows show the flow to and communication with the other cell culture microchannels. The white scale bar represents 100 µm.

Each experiment for the prospective “proof of concept” design was performed in triplicate, unless otherwise stated. The microfluidic system (shown in Figure 3A,B) was designed in AutoCAD 2021 and consisted of three main channels, which were interconnected with microchannels. The main channels had the following characteristics: channel 1 (for the U87MG culture) was 600 µm wide × 6780 µm in length × 100 µm in depth; channel 2 (for the astrocyte culture) was 200 µm wide × 16900 µm in length × 100 µm in depth; and channel 3 (for vascular cell formation) was 400 µm wide × 6750 µm in length × 100 µm in depth. The channel inlets and outlets had diameters of 2 mm. The interconnecting channels were 6 µm wide x 30 µm in height x 50 µm in length. The device was based on polydimethylsiloxane (PDMS). The microfluidic system was attached to a coverslip using corona discharge. The microfluidic system was designed at the Centro Médico Nacional ‘20 de Noviembre’ at the ISSSTE and was manufactured at Laboratorio de Micro y Nanotecnología del Laboratorio Nacional de Soluciones Biomiméticas Diagnostico y Tratamiento (LaNSBioDyT) at Universidad Nacional Autónoma de México, México City.

The permeability of the microfluidic system was evaluated as follows: 70% ethanol with blue food dye (1%, locally sourced, Mexico City, and visualized under the visible spectrum light) and Texas red dextran (10WD; Thermofisher excitation/emission wavelength at 594 nm/616 nm) was placed in every channel of the microfluidic system in order to assess microfluidic communication. In addition, dye diffusion (*D = diffusion coefficient*) was calculated (Figure 3C) considering specific parameters, like the area through which the dye diffused (according to the Fick principle), using the following equation:

$$D=\frac{x^{2}}{4t\;\left(\frac{ln\left(C\left(x/t\right)\right)}{C_{0}}\right)}$$

where *C*_0_ is the initial concentration of the dye, *C* is the concentration of the dye at a certain position and time, *x* is the distance from the initial dye location, and t is the time point of evaluation.

### 2.2. Cell Immunophenotype, Proliferation Kinetics, and Vascularization Assay

The cell lines underwent expansion in cell culture plates, according to the manufacturers’ recommendations. The cells were further seeded in 24-well plates at a density of 1 × 10^4^ cells·cm^2^. After a 72-h incubation period, the cells were fixed in 4% paraformaldehyde. Then, three washes with PBS were performed and 0.1% tween-20 was used for cell permeabilization. Subsequently, primary antibodies (Table 1) were applied, and the cells were incubated overnight at 4 °C. Next, 10% fetal bovine serum in PBS was used to block non-specific epitopes, followed by three more washes with PBS. DAPI staining was used for nuclear staining. Finally, immunopositive fluorescent cells were identified using an inverted epifluorescence microscope (Olympus IX71; Figure S1). The cellular immunophenotype was determined using the antibodies listed in Table 1.

**Figure 4:**
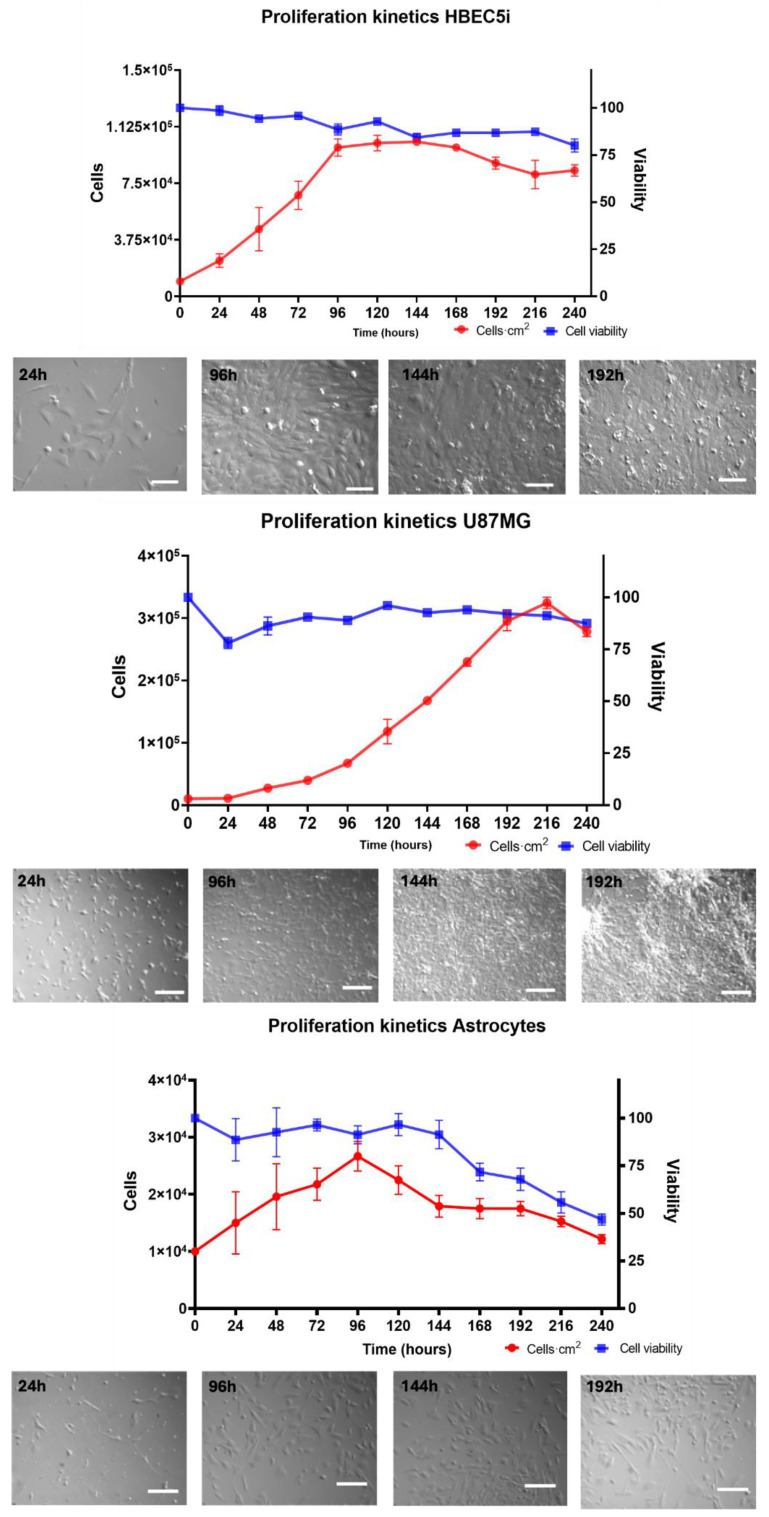
The growth kinetics of the HBEC5i (endothelial cells), U87MG (glioblastoma tumoral cells), and astrocyte cell lines. The top central graphs show the quantification of the growth rate with representative micrographs of the time course of proliferation in the culture plate below each graph. The scale bar represents 100 µm in all.

For the cell proliferation assay (Figure 4), HBEC5i, U87MG, and astrocyte cell lines were seeded in culture plates at a density of 1 × 10^4^ cells·cm^−2^ for 264 h without media replacement. Cell viability (%) was evaluated every 24 h for a maximum of 11 days (264 h) of culture, using the trypan blue 0.4% (Thermofisher 15250061, Waltham, MA, USA) exclusion test. The specific growth rate (µ) and doubling time (*T_d_*) were calculated using the following equations:

$$µ=\frac{\left(In\left(number\;of\;final\;Cells\right)-In\left(number\;of\;Original\;Cells\right)\right)\;}{\left(Final\;Time-Zero\;Time\right)}$$

Doubling time (*T_d_*):

$$T_{d}=\frac{\left(In2\right)\;}{µ}$$

### 2.3. Endothelial Vascularization Test

The ability of HBEC5i endothelial cells to form microvasculature structures was evaluated by brightfield microscopy (OLYMPUS IX71 Inverted Stand, Tokyo, Japan). For HBEC5i microvasculature induction, a protocol based on previous results from our laboratory, in which the culture medium was maintained for 96 h, was used. Briefly, HBEC5i cells were bi-dimensionally seeded at a density of 6 × 10^4^ cells·cm^−2^ on plates containing a fibrin matrix obtained from blood plasma, 0.1% gelatin, or 1:50 Matrigel (Figure S2).

### 2.4. Cell Line Cultures in the Microfluidic System

The microfluidic culture experiments were conducted between passages 3 and 6 of each cell line. The cell lines were cultured and expanded according to the technical specifications from ATCC and ScienCell. The cells were grown to 80% confluency in T25 or T75 tissue culture flasks. Subsequently, the microfluidic system inputs were optimized (Table S1) in order to achieve the best cell attachment conditions. 

**Figure 5:**
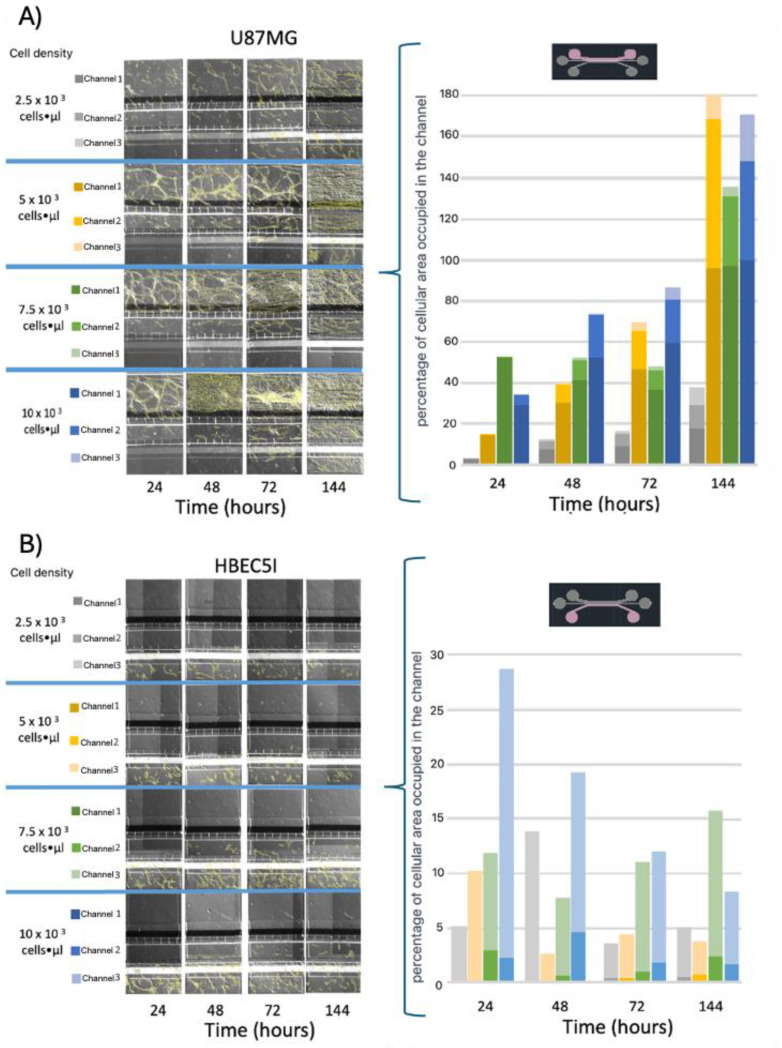
The cell migration ability of (**A**) U87MG endothelial cells and (**B**) HBEC5i at different starting cell densities. Migrating cells are indicated in yellow.

**Figure 6:**
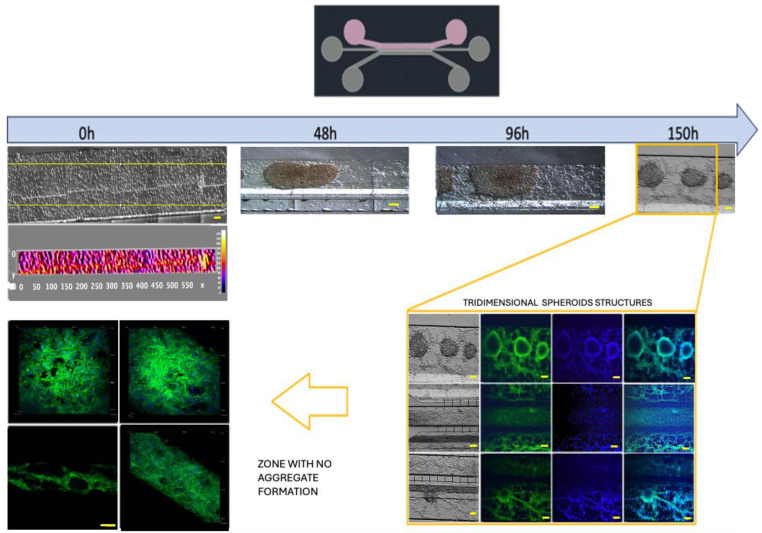
Culture U87MG in microfluidic system culture. The cell culture process at a density of 2 × 10^4^ cells·µL^−1^: a heatmap of the cell distribution at 0 h is shown for cell loading, and as time progresses (48–150 h), cell clusters that were initially formed exhibited 3D projections, which have been described as organoids, and were acquired via confocal microscopy at 150 h. The cellular cytoskeleton is shown with the expression of phalloidin (green), and the nuclei were assessed with DAPI (blue). The scale bar represents 100 µm.

**Figure 7:**
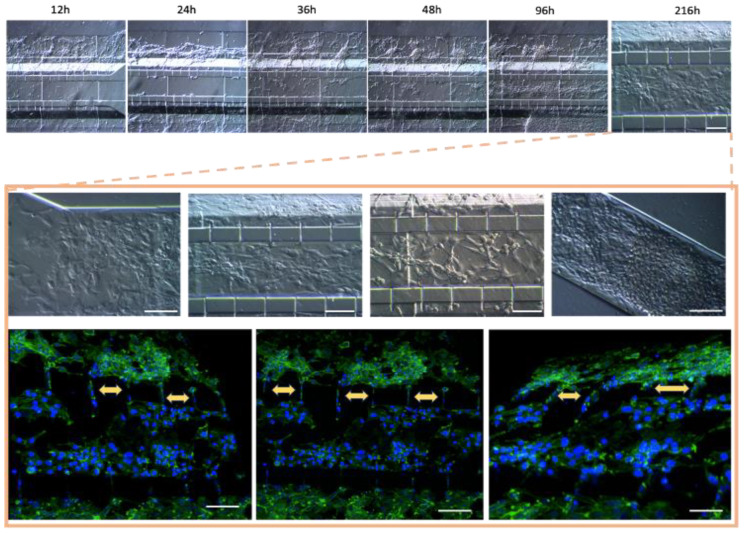
Co-culture of U87MG-HBEC5i acquired by confocal microscopy: the cell migration process. The cellular cytoskeleton is shown with the expression of phalloidin (green), and the nuclei were assessed with DAPI (blue). Cell–cell interactions are shown with yellow arrows, which occurred through interconnecting channels. The scale bar represents 100 µm.

Before cell inoculation in the microfluidic system, the devices were placed individually in Petri dishes to prevent exposure to the environment. In this enclosed system, they were irradiated with ultraviolet light for 20 min in a vertical laminar flow hood. For cell inoculation, adapted 1000-microliter micropipette tips were used to fit the inlet and outlet channels of the microfluidic system. Then, different concentrations of HBEC5i and U87MG cells were synchronically inoculated in the channels across multiple devices. The seeding densities were 2.5 × 10^3^, 5 × 10^3^, 7.5 × 10^3^, and 10 × 10^3^ cells·µL^−1^. The culture medium was replaced every 48 h (Figure 5, Figure 6, Figure 7 and Figure S3), and an optimal volume of 100 µL was determined for the maximal proliferation efficiency of HBEC5i cells within the microfluidic system (Figure S4). 

The behavior of the U87MG cell culture was assessed at a density of 2 × 10^4^ cells·µL^−1^, with daily medium changes (100 µL) for each channel. After 260 h of culture, characteristic GFAP expression in U87MG was assessed (Figure S5).

Finally, cellular detachment capability was assessed using trypsin or a mechanical air injection process (200 microliters) with 1000-microliter pipettes. This process involved cell densities of 2.5 × 10^3^, 5 × 10^3^, 7.5 × 10^3^, and 1 × 10^4^ cells·µL^−1^ (Figure S6).

### 2.5. Microenvironment of Blood–Brain Barrier

**Figure 8:**
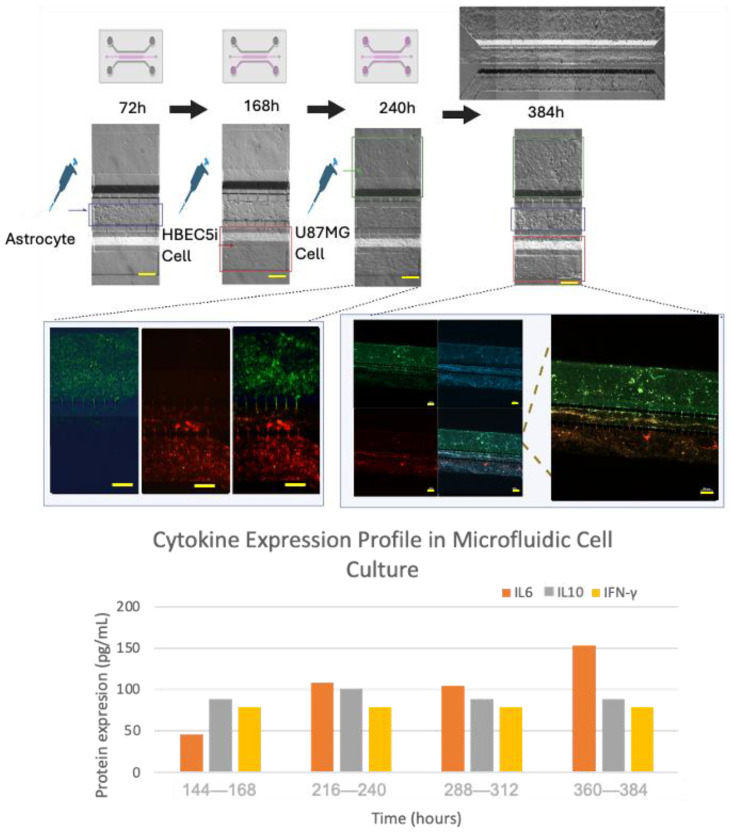
The culture evolution at different time points and the process of cell invasion. Glioblastoma U87MG cells are shown in green, endothelial HBEC5i cells are shown in red, cell nuclei are shown in blue (DAPI), and cell–cell interactions through interconnecting channels are shown by green and red arrows. The expression of cytokines was carried out by combining the triplicate model to obtain the necessary amount of culture medium (cytokine sample = 1). The scale bar represents 200 µm.

A systematic scaling of the inoculation of the cell lines within the microfluidic device was performed in order to elicit progressive cell interactions resembling the BBB. First, astrocytes were inoculated in channel 2, followed by endothelial cells being inoculated in channel 3 (labeled with Texas red dextran 10WD-D1863, Waltham, Massachusetts, USA; according to the Thermofisher instructions, with excitation/emission wavelength at 594 nm/616 nm), and finally, U87MG cells were inoculated in channel 1 (labeled with MitoTracker green according to the manufacturer’s instructions, Thermofisher -MitoTracker™ Green FM-M7514, Waltham, Massachusetts, USA). The culture medium was collected in its entirety every 24 h. The supernatants from each channel were stored separately. For subsequent analysis, the microliters from the three channels were combined into a single tube and were frozen (−80 °C) until further analyses, and culture media collected from the microfluidic system were analyzed for cytokine expression (IL-2, IL-6, IL-10, and gamma interferon) using a multiplex ELISA assay (arigoplex), following the manufacturers’ recommendations (Figure 8).

### 2.6. Confocal Microscopy

Cell lines inoculated within the microfluidic system were fixed with 4% paraformaldehyde at room temperature and washed with PBS. The antibodies and DAPI were incubated overnight. Subsequently, images were acquired at the Laboratorio Nacional de Microscopía Avanzada at the Centro Médico Nacional Siglo XXI (Instituto Mexicano del Seguro Social) and at the Centro de Desarrollo de Productos Bióticos (CEPROBI) at the Instituto Politécnico Nacional using a Nikon A1 inverted confocal microscope. The specimens were observed using a CLSM (Carl Zeiss, Model LSM800, made in Oberkochen, Germany) with a motorized stage (X, Y, Z) that was coupled to an AxioCam HD color camera (Carl Zeiss, Model 305, made in Germany). The ZEN (Zeiss efficient navigation) software (version 2.6, Blue edition) was employed. 

## 3. Results and Discussion

The present study showed that gel-free 3D-mFCCSs represent non-complex and versatile models that are able to recapitulate several features that occur at the BBB, as well as dynamic tumor cell interactions. They are comparable to hydrogel-containing microfluidic models and could offer a suitable alternative for studying CNS tumor biology. 

### 3.1. Microfluidic System Design and Characterization

In order to resemble the dynamics that occur at the BBB, a parallel microfluidic system was designed as the basic structure to support cells from different lineages, which was inspired by previously reported designs [37]. Our microfluidic system included endothelial cells, astrocytes, and tumor glioblastoma cells, in the absence of synthetic ECM. This microfluidic system demonstrated an appropriate diffusion of vegetable dye within its microchannels and their interconnections (Figure 3C) over a period of 2 min. The calculated dye diffusion coefficient of the system was 4.06 × 10^−9^ m^2^·S^−1^; however, the boundary conditions of the walls of the microfluidic channels and the shape of the microfluidic channels could affect dye diffusion [38]. Fluid distribution within a 3D-mFCCS is relevant because it ensures a leak-free system, as well as the adequate spread of the solution of interest within the microfluidic system.

### 3.2. Cellular Population and Inoculation Cell Density in the Microfluidic System

The cell populations that were considered for integration into the proposed gel-free 3D-mFCCS were those resembling the microenvironment interactions of tumor cells and the BBB, such as glioblastoma tumoral cells (U87MG), endothelial cells (HBEC5i), and astrocyte (ScienCell 1800) cell lines. The HBEC-5i cell line has advantages over other human cell lines such as TY08, TY10, HCEC, HMEC-1, BB19, HBMEC, and hCMEC/D3, which have all been extensively studied in the literature. These cell lines, particularly human cerebrovascular microvascular endothelial cells (hCMEC/D3) and human brain microvascular endothelial cells (hBMEC), are often cultured in media that contain adjuvants. These adjuvants can alter the expression of ABC transporters, impacting the relevance of findings [39]. ScienCell 1800 astrocytes represent the glial cell lineage, specifically as primary cells from the cerebral cortex. Being primary cells, they maintain their original phenotypic and functional characteristics, thereby providing a model that is more representative of real physiological conditions. These astrocytes are ideal for studies related to neurobiology, as they play key roles in maintaining neuronal environment homeostasis, in synaptic transmission, and in responses to injuries in the central nervous system [40]. Finally, the U87MG human glioblastoma cell line has a high proliferation rate, versatile applications in glioblastoma research, and is relevant to human diseases, making it a tool for studying tumor biology and therapeutic responses [41].

In contrast, the HBEC-5i cell line can grow in a monolayer without the need for adjuvants, making it an ideal model for blood–brain barrier (BBB) research.

In order to characterize morphology and cell function, in vitro assays of immunophenotype (Figure S1) and proliferation were performed in culture plates. The endothelial cells showed an average growth rate of 0.20 ± 0.0035 cells·h^−1^ and a doubling time of 37 ± 6.034 h. The U87MG cells exhibited an average growth rate of 0.018 ± 0.00070 cells·h^−1^ and a doubling time of 35.2 ± 1.98 h. As for astrocytes, their growth rate was 0.010 ± 0.00040 cells·h^−1^, with a doubling time of 67.84 ± 2.3 h (Figure 4). An analysis of the maintenance of immunophenotype and vasculogenic (Figure S2) ability was also performed. Notably, the U87MG cells showed the highest duplication rate, followed by endothelial cells and astrocytes. Therefore, these cellular lineages exhibited natural behaviors. It is worth mentioning that astrocytes constitute a primary cell culture line, whereas HBEC5i is an immortalized cell line. Despite this distinction, the HBEC5i cell line displayed functional characteristics that were remarkably like those of physiological endothelial cells, as observed in the expression of endothelial cell proteins and their capacity for microvascular formation, thereby representing a valuable cellular tool for in vitro studies (Figure S2B). Additionally, other research groups have used these cells as models for brain cell interactions and have proved them to be more suitable than the implementation of HUVEC cells or other endothelial cell lines [39]. On the other hand, the U87MG cells showed the expression of Oct3/4 and Sox2, which is characteristic of cancer stem cells, as well as the expression of GFAP.

### 3.3. Optimization of Inoculation Cell Density in the Microfluidic System 

Similar to hydrogel-containing microfluidic systems, gel-free 3D-mFCCSs allow for the study of different cellular processes, such as growth, adaptability, migration, and cell interactions. For this purpose, the cells within the proposed gel-free microfluidic system had to be arranged in layers, and several optimization approaches were performed to achieve the final optimal working conditions (Table S1; Figure S7). In the present study, these optimal conditions included 100 microliters of culture medium, which contained the cell lines at the cell concentration indicated for each experiment and inoculated in the corresponding channel reservoir (Figure S4). The inoculation of the individual cell lines in the device and the filling of all channels with culture medium induced cell migration from one channel to the other.

Further optimization methods included U87MG cells being used at a cell density of 1 × 10^4^ cells·µL^−1^ to achieve uniform distribution and cell growth within the 3D-mFCCS (Figure 5), as well as a cell density of 2 × 10^4^ cells·µL^−1^ for 3D growth, which resembled tumor organoids (Figure 6 and Figure S5). Meanwhile, the cell interactions between HBEC5i and U87MG were optimized via co-culture at a cell density of 1 × 10^4^ cells·µL^−1^ (Figure 6). Of note, the higher cell densities of the U87MG line resulted in the formation of three-dimensional clusters, which have been described as organoids in other reports. Such organoid structures do not exhibit contact inhibition and resemble a tumor shape within similar microenvironments. The migration process of U87MG in the microfluidic system was successfully visualized using confocal microscopy. Interestingly, the U87MG cells (with a diameter range of 12 to 16 μm) modified their cellular structure to migrate through the 6-micrometer channel, as shown in Figure 7 and Figure S8. 

In order to recreate an appropriate BBB architecture in our gel-free 3D-mFCCS model, we ensured that different cell strains did not mix but instead proliferated individually within their respective channels, as illustrated in Figure S8. For this purpose, the cultured HBEC5i cells were inoculated first, followed by the U87MG cells, and then the astrocytes. This inoculation order facilitated communication between the HBEC5i and U87MG cells (Figure 8).

The optimization of microfluidic systems and cell integration within gel-free 3D-mFCCSs is a key point for developing appropriate models of the BBB. Some adequations may be performed by applying a constant pressure of culture medium through microfluidic systems [41]. On the other hand, the application of shear stress may stimulate the mechanoreceptors on endothelial cells and influence various cellular functions, including cytoskeletal remodeling, gene expression, cell viability, and calcium homeostasis, thereby controlling the myogenic tone [42]. Shear stress may also affect the formation of tight junctions, leading to selective permeability and improvement of the barrier function [18,43,44]. Understanding shear stress-mediated changes in transporter activity is vital for designing effective drug delivery strategies that target the CNS. The dysregulation of shear stress responses in endothelial cells is implicated in the pathogenesis of conditions in the CNS. Understanding the interplay between shear stress and BBB dysfunction in these diseases could offer insights into novel therapeutic interventions [28].

### 3.4. Cell–Cell Interactions within the Microfluidic System and Pro-Inflammatory Mediators

Once the different cell lines were inoculated and arranged within our gel-free 3D-mFCCS, the cell-to-cell interactions between the glioblastoma cells (U87MG) and the brain endothelial cells (HEBC5i) were confirmed (Figure 7 and Figure 8). These findings suggested that the gel-free 3D-mFCCS promoted interactions between the glioblastoma and endothelial cells, which mimicked tumor microenvironments in terms of cell–cell interactions. In this sense, cellular interactions within a microfluidic system are relevant since they are a requirement for the maturation of microvasculature, as reported by Deosarkar et al. (2015) [45]. 

Furthermore, the cell interactions within our microfluidic system correlated with the production of pro-inflammatory mediators, as shown by the three-fold increase in IL6 that accompanied the organized cell interactions after 360–384 h of culture (Figure 8), suggesting that the proposed gel-free 3D-mFCCS model was able to mimic endothelial changes that occur at the BBB during inflammatory processes. Likewise, the tumor cell proliferation and invasion also correlated with the three-fold increase in the expression of IL6 and IL10, showing a slight fluctuation in the case of interferon gamma. 

Similarly, the humanized BBB microfluidic model integrated primary human brain-derived microvascular endothelial cells and cultured pericytes and astrocytes, and was able to describe changes in metabolites and cytokines in response to inflammation, which are related to disruption of BBB permeability [30]. Fluctuations in IL10 and interferon gamma are processes that are described in tumor microenvironments, where IL6 facilitates the disruption of tight junctions in endothelial cells, allowing mobilization in the vasculature. 

### 3.5. Cell Detachment in the Microfluidic System 

Cell detachment from 3D-mFCCSs has demonstrated an independent effect for each treated channel, particularly under the conditions of low cell densities. This phenomenon highlights the capacity of cells to retain biochemical communication and cellular contact despite being detached from the substrate. Such findings suggest that gel-free models may offer significant methodological advantages over traditional models that utilize synthetic extracellular matrices (ECMs) in microfluidic systems.

In particular, the ability of cells to detach while maintaining communication implies more dynamic interactions with their environments, allowing for the better simulation of in vivo conditions. This characteristic could enhance experimental reproducibility and flexibility as researchers can manipulate cell behaviors without the constraints imposed by synthetic ECMs. Furthermore, the observed independence of detachment effects across channels indicates that researchers can tailor specific conditions for each channel, potentially leading to more nuanced insights into cellular responses and interactions.

By utilizing gel-free 3D-mFCCSs, researchers may be able to explore a broader range of cellular behaviors and interactions, ultimately leading to a more comprehensive understanding of cellular dynamics in various biological contexts. The implications of these findings underscore the potential of gel-free models to advance microfluidic technologies in cell cultures, particularly for applications in tissue engineering, drug testing, and personalized medicine. This methodological innovation may pave the way for future studies aiming to elucidate the complex relationships between cells and their microenvironments, as illustrated in Figure S6.

## 4. Conclusions

In the future, microfluidic devices in glioblastoma research will include advancements in biomimetic models, technological improvements in gel-free 3D-mFCCSs regarding the incorporation of multiple cell types, and the integration of imaging and sensing technologies for continuous monitoring, in order to develop personalized medicine and to further therapeutic research [25,26]. 

The development of microfluidic devices combining resembling structural designs, appropriate ECMs, and cell interactions that closely mimic BBB microenvironments presents many challenges. The proposed gel-free 3D-mFCCS represents a versatile and suitable system that promotes cell interactions between two or three cell lines and mimics tumor–BBB microenvironments, even during inflammatory responses.

## Figures and Tables

**Table 1:** Antibodies used for phenotypic characterization.

Antibody	Primary Antibody	Secondary Antibody	Target Description
Anti Ve-cadherin	1:200 (Ab33168)	1:1000 (Ab6718)	Ve-cadherin is an adhesion protein that is present in endothelial cells that form the inner lining of blood vessels.
Anti von Willebrand	1:200 (Ab6994)	1:1000 (Ab6718)	The Von Willebrand protein is mainly found in endothelial cells that line blood vessels.
Anti VEGFR	1:200 (ab9530)	1:1000 (ab6786)	The VEGFR (vascular endothelial growth factor receptor) is a protein that plays a crucial role in angiogenesis and blood vessel growth.
Anti Oct3/4	1:200 (C10-Z210Ms)	1:1000 (ab6786)	The expression of Oct3/4 in glioblastoma cells, such as U87MG, is an indication of these tumor cells’ ability to acquire more undifferentiated and aggressive characteristics.
Anti Sox2	1:200 (ZM57-Z236MS)	1:1000 (ab6786)	Sox2 expression in glioblastoma cells, including U87MG, signals the existence of stem cells that are capable of self-renewal and the generation of differentiated tumor cell subpopulations.
Anti GFAP	1:200 (G3893)	1:1000 (ab6785)	GFAP (glial fibrillary acidic protein) can be used for identifying and locating astrocytes in cell cultures, brain tissues, and histological sections.
Anti Phalloidin-Alexa- Fluor 488	1:100 (A12379)	-------------	The anti-phalloidin antibody can be used to visualize the distribution, organization, and dynamics of actin filaments in living cells or fixed samples.
Anti Claudin 5	1:50 (Ab15106)	1:1000 (ab6785)	Claudin 5 is a protein that is expressed in the tight junctions of endothelial cells.
Anti ZO1	1:50 (Ab221547)	1:1000 (Ab6717)	Zonula occludens-1 is a tight junction protein that plays a key role in the formation and maintenance of tight cellular junctions.

## Data Availability

The original contributions presented in the study are included in the article/Supplementary Materials, further inquiries can be directed to the corresponding author.

## Supplementary Materials

The following supporting information is available online at https://www.mdpi.com/article/10.3390/bioengineering11101008/s1, Table S1: Approach strategies for cell cultivation within microfluidic systems, Figure S1: The immunophenotype characterization of HBEC5i (endothelial cells) and U87MG (glioblastoma tumoral cells) cell lines in a culture plate. Figure S2: The vasculogenesis ability of HBEC5i: (a) the optimization of cell maintenance for HBEC5i cells; (b) vasculogenic assay; and (c) vasculogenic structure on a gelatin matrix after 96 h of culture. Initial cell culture = 6 × 10^4^ cells·cm^−2^. Figure S3. Cellular maintenance within the microfluidic system. In the upper image, different densities of U87MG (inoculated in the microfluidic system) and their maintenance over a period of 144 h are shown. In the lower image, different densities of HBEC5i (inoculated in the microfluidic system) and their maintenance over a period of 144 h are shown. Figure S4. Cell detachment was evaluated at various cell densities using the trypsinization process conducted over a 5–20 min standard incubation period. In the lower image, mechanical detachment via air injection is demonstrated. Figure S5. The influence of cell culture medium on maintenance and proliferation in the devices. Two different volumes of each cell culture medium were evaluated with constant changes every day. From 24 to 120 h, 50 microliters of culture medium were used, while from 144 to 172 h, the volume was increased to 100 microliters. Figure S6. Glial fibrillary acidic protein (GFAP) expression in U87MG microfluidic systems. Following the seeding of U87MG cells in the microfluidic system, the expression of GFAP was assessed within the device (indicated in green), with cell nuclei stained in blue (DAPI). Figure S7. Strategies employed for proper cellular adhesion within the microfluidic system: (a) surface view of the microfluidic system, with and without steps (left and right sides, respectively); (b) side view of the devices, with and without steps (left and right sides, respectively); (c) adaptation of medical-grade silicone tubing at each entry and exit point of the device; (d) longer adapted tubes using a clamping system; (e) adaptation of the microfluidic system with 1000-microliter pipette tips as reservoirs for the culture medium; (f) adaptation of the microfluidic system with 200-microliter pipette tips as reservoirs for the culture medium; and (g) representative results showing poor cell adhesion and bubble generation. Figure S8. Brain tumor microenvironment. F-actin expression is shown in green (Phalloidin) and cell nuclei are stained in blue (DAPI).

## References

[1] Cucullo L., Oby E., Hallene K., Aumayr B., Rapp E., Janigro D., Dermietzel R., Spray D.C., Nedergaard M. (2006). Artificial blood-Brain Barriers. Blood-Brain Barriers: FROM Ontogeny to Artificial Interfaces.

[2] Marchi N., Banjara M., Janigro D. (2016). Blood-brain barrier, bulk flow, and interstitial clearance in epilepsy. J. Neurosci. Methods.

[3] Hacking R., Hunt K. (2007). Cerebral abscess: A review of its pathophysiology, diagnosis and management. Br. J. Neurosci. Nurs..

[4] Lecuyer M.A., Kebir H., Prat A. (2016). Glial influences on BBB functions and molecular players in immune cell trafficking. Biochim. Biophys. Acta (BBA)-Mol. Basis Dis..

[5] Vatine G.D., Barrile R., Workman M.J., Sances S., Barriga B.K., Rahnama M., Barthakur S., Kasendra M., Lucchesi C., Kerns J. (2019). Human iPSC-derived blood-brain barrier chips enable disease modeling and personalized medicine applications. Cell Stem Cell.

[6] Wang X., Hou Y., Ai X., Sun J., Xu B., Meng X., Zhang Y., Zhang S. (2020). Potential applications of microfluidics-based blood-brain barrier (BBB)-on-chips for in vitro drug development. Biomed. Pharmacother..

[7] Furnari F.B., Fenton T., Bachoo R.M., Mukasa A., Stommel J.M., Stegh A., Hahn W.C., Ligon K.L., Louis D.N., Brennan C. (2007). Malignant astrocytic glioma: Genetics, biology, and paths to treatment. Genes Dev..

[8] Junttila M.R., De Sauvage F.J. (2013). Influence of tumour micro-environment heterogeneity on therapeutic response. Nature.

[9] Becker J.C., Andersen M.H., Schrama D., thor Straten P. (2013). Immune-suppressive properties of the tumor microenvironment. Cancer Immunol. Immunother..

[10] Charles N.A., Holland E.C., Gilbertson R., Glass R., Kettenmann H. (2012). The brain tumor microenvironment. Glia.

[11] Meacham C.E., Morrison S.J. (2013). Tumour heterogeneity and cancer cell plasticity. Nature.

[12] Gronseth E., Wang L., Harder D.R., Ramchandran R. (2018). The Role of Astrocytes in Tumor Growth and Progression. Astrocyte-Physiology and Pathology.

[13] Lee G., Hall R.R., Ahmed A.U. (2016). Cancer stem cells: Cellular plasticity, niche, and its clinical relevance. J. Stem Cell Res. Ther..

[14] Abou-Antoun T.J., Hale J.S., Lathia J.D., Dombrowski S.M. (2017). Brain cancer stem cells in adults and children: Cell biology and therapeutic implications. Neurotherapeutics.

[15] Ma YH V., Middleton K., You L., Sun Y. (2018). A review of microfluidic approaches for investigating cancer extravasation during metastasis. Microsyst. Nanoeng..

[16] Ruiz-Garcia H., Alvarado-Estrada K., Schiapparelli P., Quinones-Hinojosa A., Trifiletti D.M. (2020). Engineering three- dimensional tumor models to study glioma cancer stem cells and tumor microenvironment. Front. Cell. Neurosci..

[17] Yissachar N., Zhou Y., Ung L., Lai N.Y., Mohan J.F., Ehrlicher A., Weitz D.A., Kasper D.L., Chiu I.M., Mathis D. (2017). An intestinal organ culture system uncovers a role for the nervous system in microbe-immune crosstalk. Cell.

[18] Elbakary B., Badhan R.K. (2020). A dynamic perfusion based blood-brain barrier model for cytotoxicity testing and drug permeation. Sci. Rep. (Nat. Publ. Group).

[19] van Duinen V., Trietsch S.J., Joore J., Vulto P., Hankemeier T. (2015). Microfluidic 3D cell culture: From tools to tissue models. Curr. Opin. Biotechnol..

[20] Plummer S., Wallace S., Ball G., Lloyd R., Schiapparelli P., Quiñones-Hinojosa A., Hartung T., Pamies D. (2019). A Human iPSC-derived 3D platform using primary brain cancer cells to study drug development and personalized medicine. Sci. Rep..

[21] Tsai H.F., Trubelja A., Shen A.Q., Bao G. (2017). Tumour-on-a-chip: Microfluidic models of tumour morphology, growth and microenvironment. J. R. Soc. Interface.

[22] Meng F., Meyer C.M., Joung D., Vallera D.A., McAlpine M.C., Panoskaltsis-Mortari A. (2019). 3D Bioprinted In Vitro Metastatic Models via Reconstruction of Tumor Microenvironments. Adv. Mater..

[23] Zhang H., Zhu Y., Shen Y. (2018). Microfluidics for cancer nanomedicine: From fabrication to evaluation. Small.

[24] Logun M., Zhao W., Mao L., Karumbaiah L. (2018). Microfluidics in malignant glioma research and precision medicine. Adv. Biosyst..

[25] Cai X., Briggs R.G., Homburg H.B., Young I.M., Davis E.J., Lin Y.-H., Battiste J.D., Sughrue M.E. (2020). Application of microfluidic devices for glioblastoma study: Current status and future directions. Biomed Microdevices.

[26] Prabhakarpandian B., Shen M.C., Nichols J.B., Mills I.R., Sidoryk-Wegrzynowicz M., Aschner M., Pant K. (2013). SyM-BBB: A Microfluidic Blood Brain Barrier Model. Lab A Chip.

[27] Cucullo L., Hossain M., Rapp E., Manders T., Marchi N., Janigro D. (2007). Development of a humanized in vitro blood-brain barrier model to screen for brain penetration of antiepileptic drugs. Epilepsia.

[28] Cucullo L., Hossain M., Puvenna V., Marchi N., Janigro D. (2011). The role of shear stress in Blood-Brain Barrier endothelial physiology. BMC Neurosci..

[29] Herland A., van der Meer A.D., FitzGerald E.A., Park T.E., Sleeboom J.J., Ingber D.E. (2016). Distinct Contributions of Astrocytes and Pericytes to Neuroinflammation Identified in a 3D Human Blood-Brain Barrier on a Chip. PLoS ONE.

[30] Brown J.A., Codreanu S.G., Shi M., Sherrod S.D., Markov D.A., Neely M.D., Britt C.M., Hoilett O.S., Reiserer R.S., Samson P.C. (2016). Metabolic consequences of inflammatory disruption of the blood-brain barrier in an organ-on-chip model of the human neurovascular unit. J. Neuroinflamm..

[31] Maoz B.M., Herland A., FitzGerald E.A., Grevesse T., Vidoudez C., Pacheco A.R., Sheehy S.P., Park T.E., Dauth S., Mannix R. (2018). A linked organ-on-chip model of the human neurovascular unit reveals the metabolic coupling of endothelial and neuronal cells. Nat. Biotechnol..

[32] Brown J.A., Faley S.L., Shi Y., Hillgren K.M., Sawada G.A., Baker T.K., Wikswo J.P., Lippmann E.S. (2020). Advances in blood-brain barrier modeling in microphysiological systems highlight critical differences in opioid transport due to cortisol exposure. Fluids Barriers CNS.

[33] Park T.-E., Mustafaoglu N., Herland A., Hasselkus R., Mannix R., FitzGerald E.A., Prantil-Baun R., Watters A., Henry O., Benz M. (2019). Hypoxia-enhanced Blood-Brain Barrier Chip recapitulates human barrier function and shuttling of drugs and antibodies. Nat. Commun..

[34] Morad G., Carman C.V., Hagedorn E.J., Perlin J.R., Zon L.I., Mustafaoglu N., Park T.-E., Ingber D.E., Daisy C.C., Moses M.A. (2019). Tumor-Derived Extracellular Vesicles Breach the Intact Blood-Brain Barrier via Transcytosis. ACS Nano.

[35] Goral V.N., Hsieh Y.C., Petzold O.N., Clark J.S., Yuen P.K., Faris R.A. (2010). Perfusion-based microfluidic device for three-dimensional dynamic primary human hepatocyte cell culture in the absence of biological or synthetic matrices or coagulants. Lab A Chip.

[36] Gupta N., Liu J.R., Patel B., Solomon D.E., Vaidya B., Gupta V. (2016). Microfluidics-based 3D cell culture models: Utility in novel drug discovery and delivery research. Bioeng. Transl. Med..

[37] Jeon J.S., Bersini S., Gilardi M., Dubini G., Charest J.L., Moretti M., Kamm R.D. (2015). Human 3D vascularized organotypic microfluidic assays to study breast cancer cell extravasation. Proc. Natl. Acad. Sci. USA.

[38] Puech C., Hodin S., Forest V., He Z., Mismetti P., Delavenne X., Perek N. (2018). Assessment of HBEC-5i endothelial cell line cultivated in astrocyte conditioned medium as a human blood-brain barrier model for ABC drug transport studies. Int. J. Pharm..

[39] Gordillo-Sampedro S., Antounians L., Wei W., Mufteev M., Lendemeijer B., Kushner S.A., de Vrij FM S., Zani A., Ellis J. (2024). iPSC-derived healthy human astrocytes selectively load miRNAs targeting neuronal genes into extracellular vesicles. Mol. Cell. Neurosci..

[40] Boccellato C., Rehm M. (2022). Glioblastoma, from disease understanding towards optimal cell-based in vitro models. Cell. Oncol..

[41] Arvanitis C.D., Ferraro G.B., Jain R.K. (2020). The blood–brain barrier and blood–tumour barrier in brain tumours and metastases. Nat. Rev. Cancer.

[42] Tovar-Lopez F., Thurgood P., Gilliam C., Nguyen N., Pirogova E., Khoshmanesh K., Baratchi S. (2019). A microfluidic system for studying the effects of disturbed flow on endothelial cells. Front. Bioeng. Biotechnol..

[43] Adriani G., Ma D., Pavesi A., Kamm R.D., Goh E.L. (2017). A 3D neurovascular microfluidic model consisting of neurons, astrocytes and cerebral endothelial cells as a blood–brain barrier. Lab A Chip.

[44] May S., Evans S., Parry L. (2017). Organoids, organs-on-chips and other systems, and microbiota. Emerg. Top. Life Sci..

[45] Deosarkar S.P., Prabhakarpandian B., Wang B., Sheffield J.B., Krynska B., Kiani M.F. (2015). A novel dynamic neonatal blood-brain barrier on a chip. PLoS ONE.

