# Supplementary material for: A Versatile Microfluidic Device System that Lacks a Synthetic Extracellular Matrix Recapitulates the Blood–Brain Barrier and Dynamic Tumor Cell Interaction
Source: Bioengineering (Basel). 2024 Oct 10;11(10):1008. doi: 10.3390/bioengineering11101008 (PMC11505467; doi:10.3390/bioengineering11101008)
Supplement: Supplementary file 1 [file bioengineering-11-01008-s001.zip › bioengineering-3187905-supplementary.pdf]

Table S1. Approach strategies for cell cultivation within microfluidic systems.

Supplementary Table 1. Approach Strategies for Cell Cultivation within Microfluidic Systems

**Microfluidic Device**	**Evaluated cells**	**Detected difficulties**
Inlet-outlet (1mm)	HBEC5i cell density of 6000 in 20 µL<br>HBEC5i cell density of 10000 in 20 µ	Evaporation of culture medium within 24 hours<br>Cellular stress<br>Lack of cell adhesion<br>Cell death<br>Established population at the edges
Inlet-outlet (2mm)	HBEC5i cell density of 10000 in 20 µL	Low cell population adhesion<br>2-days cellular population maintenance
Inlet-outlet (1mm) + Step	HBEC5i cell density of 10000 in 20 µL	- Low cell population adhesion<br>- 2-days cellular population maintenance
Inlet-outlet (2mm) + Step	HBEC5i/U87MG cell density of 10000 in 20 µL	- 3-days cellular population maintenance
Silicone tube 10 cm	HBEC5i/U87MG cell density of 10000 in 20 µL	-Low adhesion of the cell population<br>- Cellular stress and death
Silicone tube 0.5 cm	HBEC5i/U87MG cell density of 10000 in 20 µL	- Low adhesion of the cell celular<br>- Cellular stress and death<br>- Established population at the edges
Adapted 200 µL tip in Inlet-outlet (2mm) + Step	HBEC5i/U87MG cell density of 50000 in 20 µL	- Proper cell adhesion
Adapted 1000 µL tip in Inlet-outlet (2mm) + Step	HBEC5i/U87MG cell density of 50000 in 20 µL	- Proper cell adhesion

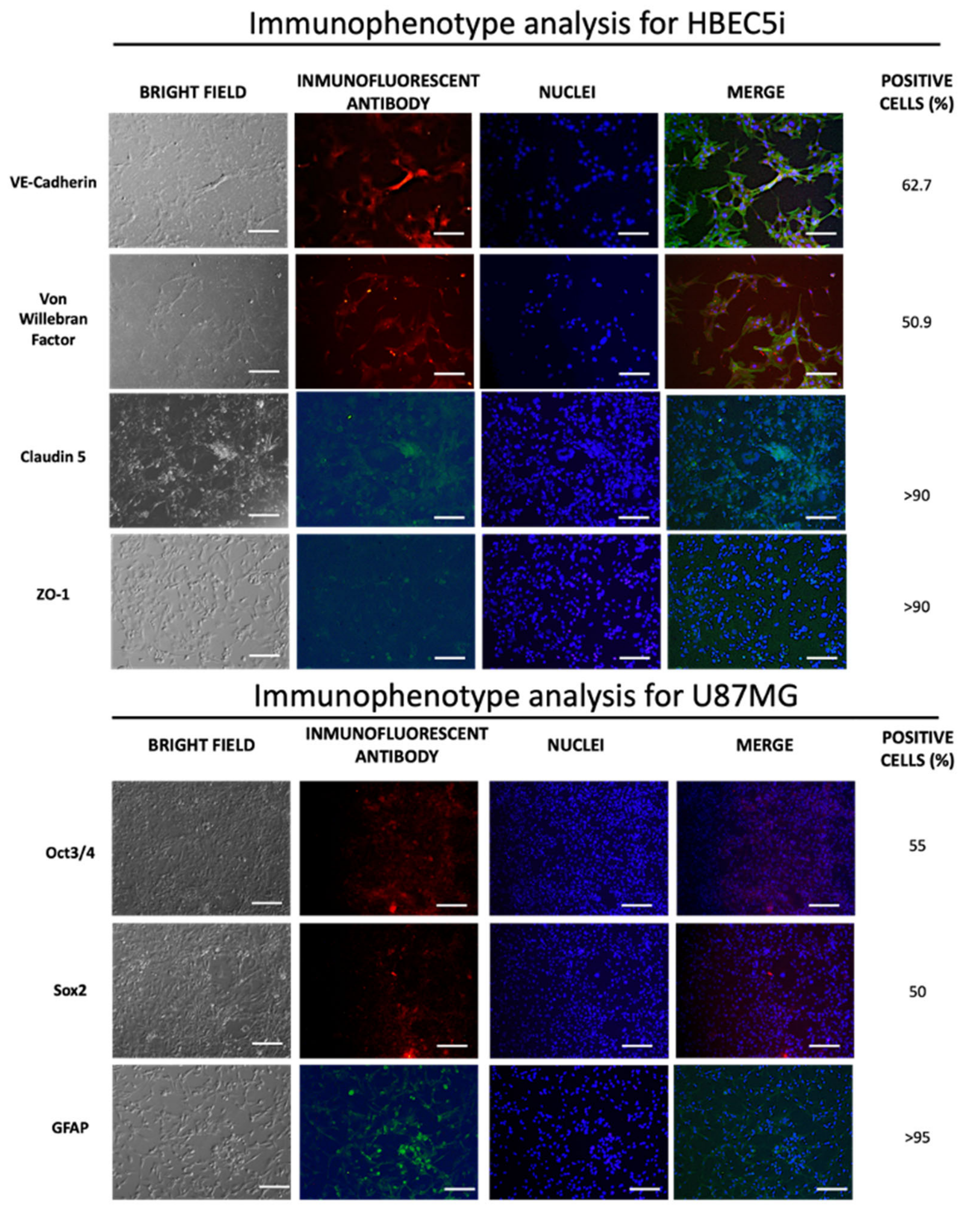

Figure S1. Immunophenotype characteristics. The figure shows immunophenotype characterization of HBEC5i (endothelial cells) and U87MG (glioblastoma tumoral cells) cell lines in culture plate; as indicated by images of bright field, immunofluorescence, DAPI and merge. Percentage of microscope field positive for immunostaining is also showed. The withe scale bar represents 100 μm.

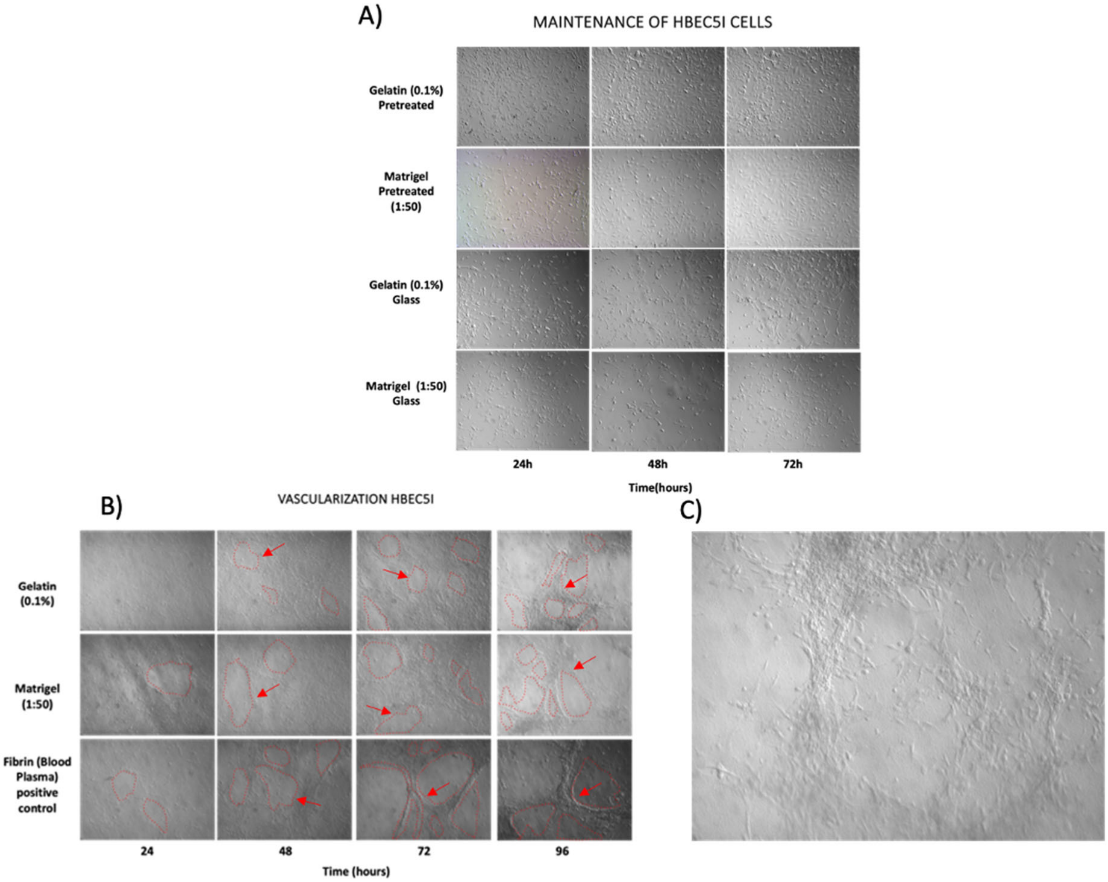

Figure S2. Vasculogenesis ability of HBEC5i. A) Optimization of cell maintainance for HBEC5i cells. B) Vasculogenic assay. C) Vasculogenic structure on gelatin matrix at 96 hours culture. The figure shows the ability of endothelial cells HBEC5i to generate vascular shapes, regardless the matrix used. Cell seeding concetration was 6 x 10^4 cells•cm $^{-2}$.

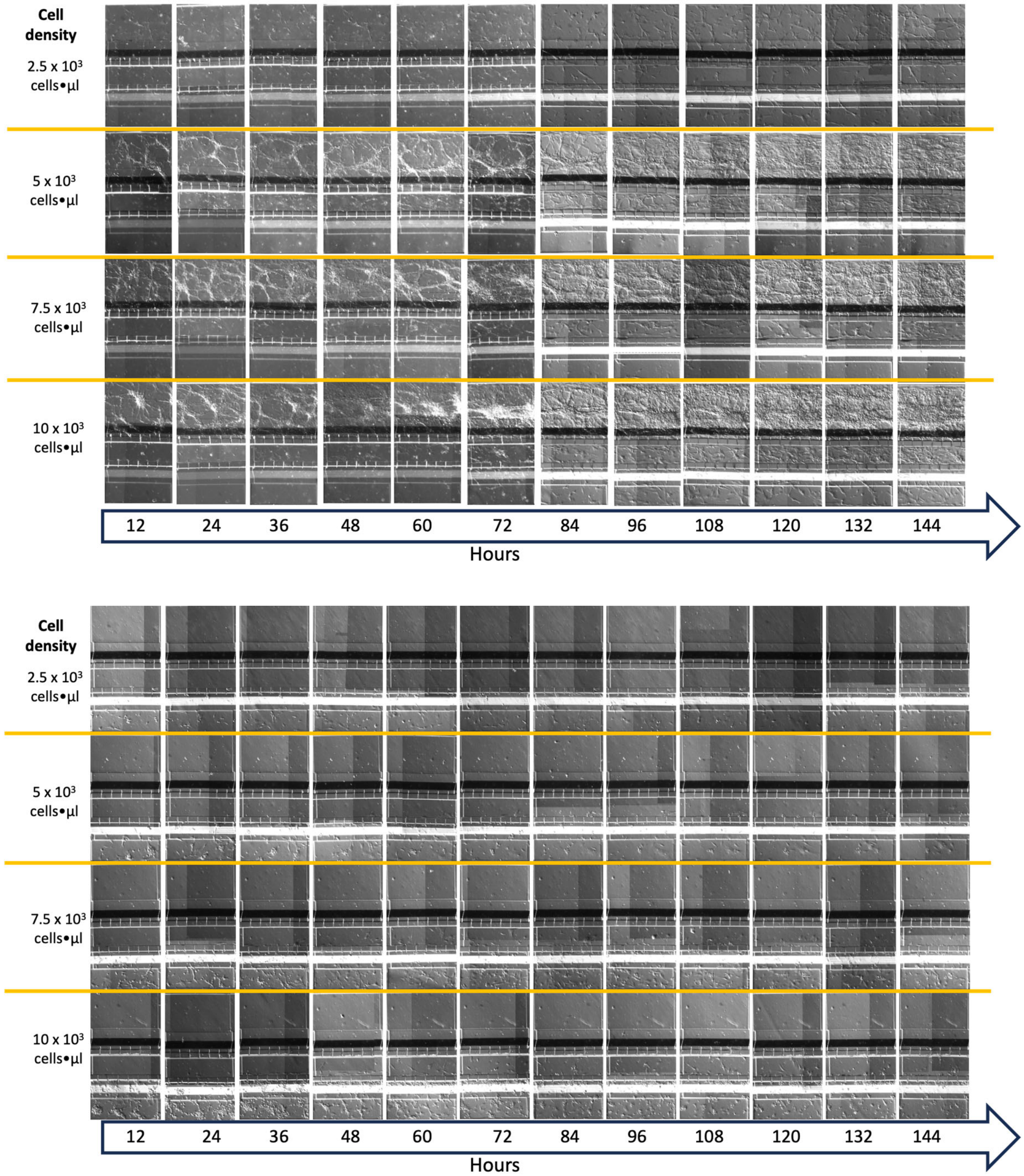

Figure S3. Cell culture maintenance within the microfluid system. The upper image shows the different densities of U87MG inoculated in the microfluid system and its maintenance over a period of 144h. Lower image shows the different densities of HBEC5i inoculated in the micro fluid system and its maintenance in a period of 144 hours.

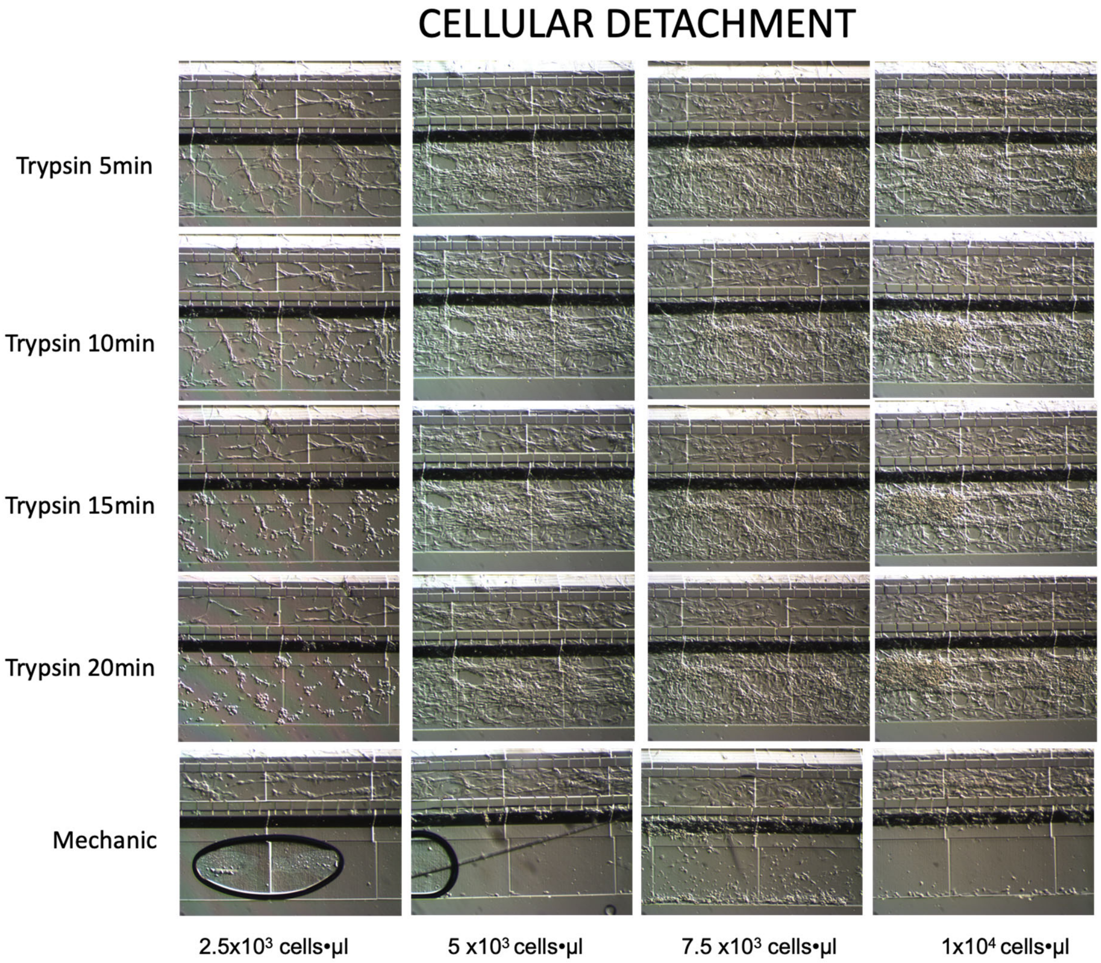

Figure S4. Cell detachment. Cell detachment was evaluated at various cell densities using the trypsinization process conducted over a 5-20 minute standard incubation period. At the bottom, mechanical detachment via air injection is demonstrated.

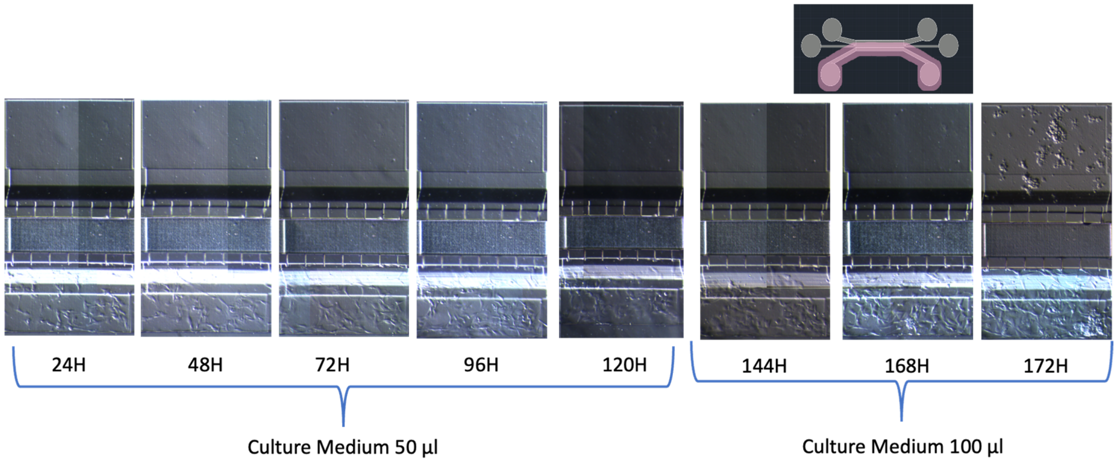

Figure S5. Influence of cell culture medium on maintenance and proliferation in the devices. Two quantities of culture medium were evaluated with constant changes every day. From 24-120 hours, 50 microliters of culture medium were used, while from 144-172 hours, the volume was increased to 100 microliters.".

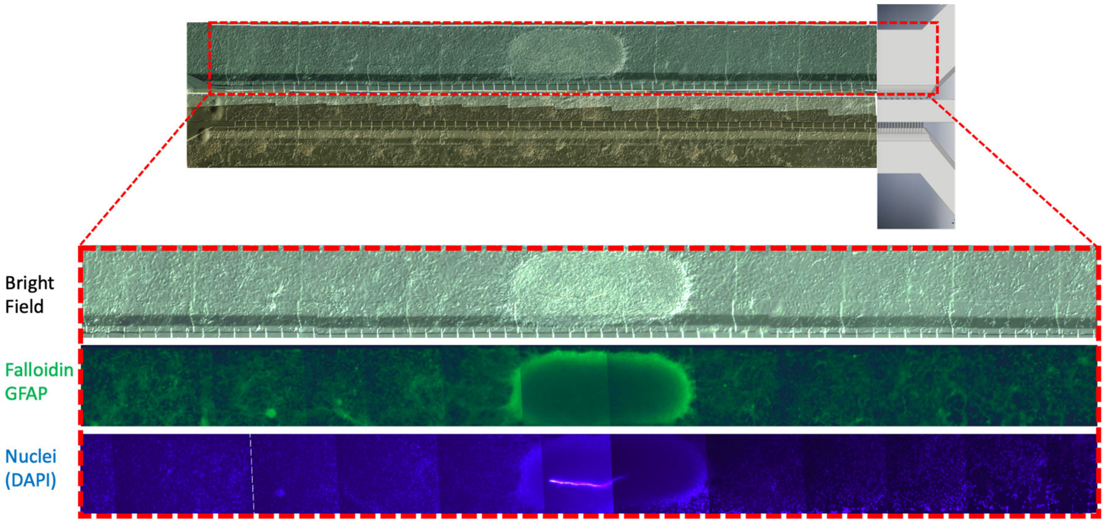

Figure S6. Glial Fibrillary Acidic Protein (GFAP) expression in U87MG microfluidic systems. Following the seeding of U87MG cells in the microfluidic system, the expression of GFAP was assessed within the device (green), with cell nuclei stained in blue (DAPI).

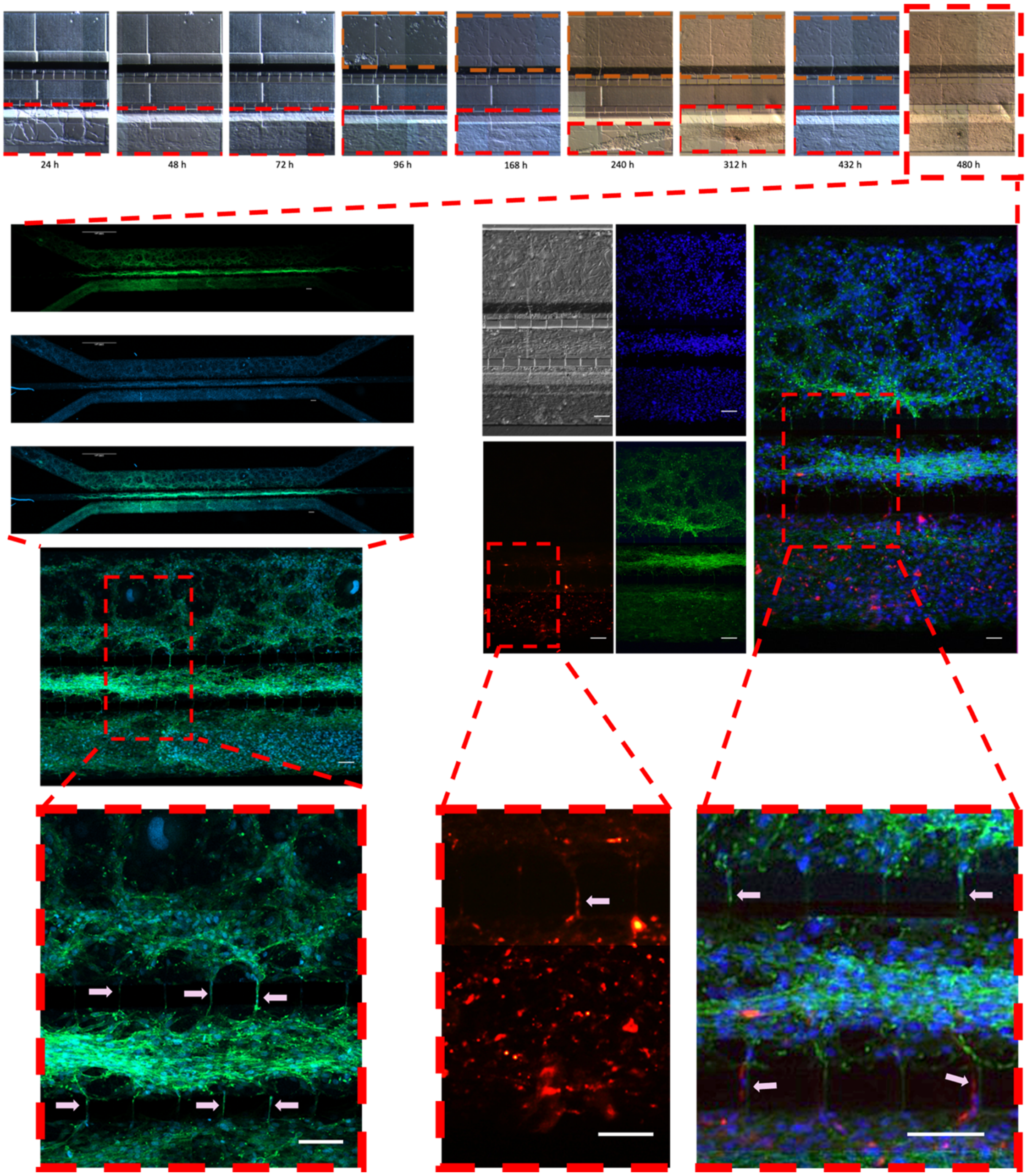

Figure S7. Brain Tumor cell Microenvironment. The temporal scaling of cell culture is observed, starting with the inoculation of HBEC5i cells (labeled with Texas Red Dextran). Subsequently, cells from the opposite end (U87MG) were introduced, and finally, astrocyte cells were inoculated. F-actin expression is shown in green (Phalloidin), and cell nuclei are stained in blue (DAPI). In the lower below left Figure, cell migration trough communication channels was observed (arrow).

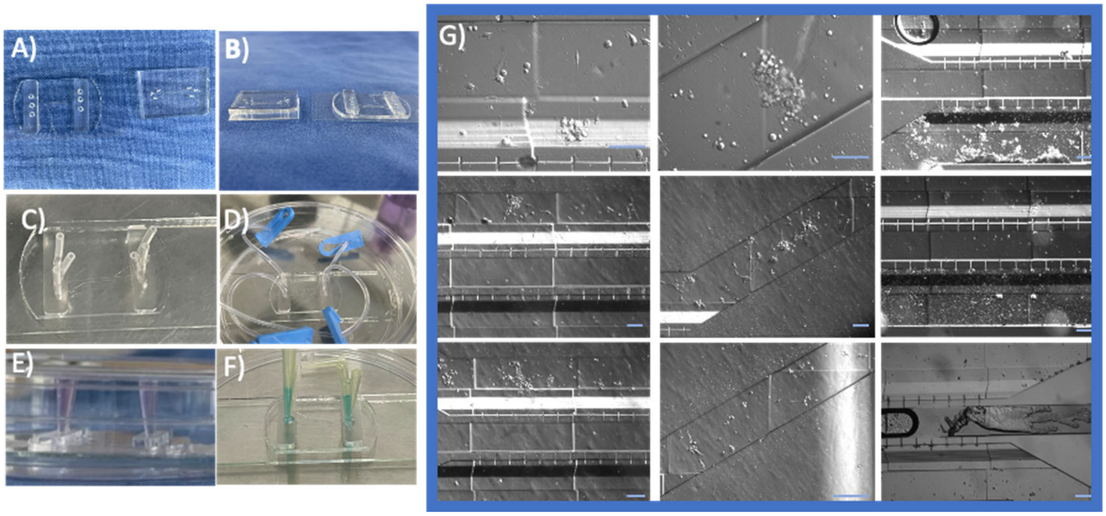

Figure S8. Strategies employed for proper cellular adhesion within the microfluidic system. A) Surface view of the microfluidic system with and without steps (left and right sides, respectively), B) Side view of the devices on the right (with steps) and left (without steps), C) Adaptation of medical-grade silicone tubing at each entry and exit of the device, D) Longer adapted tubes using a clamping system, E) Adaptation of the microfluidic system with thousand-microliter pipette tips as a reservoir for culture medium, F) Adaptation of the microfluidic system with two-hundred-microliter pipette tips as a reservoir for culture medium, and G) Representative results showing poor cell adhesion, bubble generation.
